# Radiological Evaluation of Retained Primary Molars in Adolescents with Mandibular Second Premolar Agenesis

**DOI:** 10.3390/jcm14093241

**Published:** 2025-05-07

**Authors:** Dita Meistere, Aleksandra Karkle, Sindija Mengele, Liga Kronina, Laura Neimane

**Affiliations:** 1 Department of Conservative Dentistry and Oral Health, Riga Stradins University, LV1007 Riga, Latvia; 2 Institute of Stomatology, Riga Stradins University, LV1007 Riga, Latvia

**Keywords:** tooth agenesis, infraocclusion, root resorption, panoramic X-ray, hypodontia

## Abstract

**Objectives**: We aimed to evaluate the condition of retained primary molars in case of mandibular second premolar agenesis. **Materials and Methods**: In total, 2692 panoramic radiographs of adolescent patients were analyzed to determine the prevalence of tooth agenesis. Patients (N = 156) with at least one mandibular second premolar agenesis were further explored to determine the presence and condition of retained primary teeth. Teeth were classified as good or poor based on root resorption, infraocclusion, caries, and restorations. **Results**: The prevalence of mandibular second premolar agenesis was 5.8% (N = 156). For the set period for this study, 138 (58.5%) primary molars were present. Out of these retained teeth, N = 83 were in good condition. There was a statistically significant positive weak correlation between age and the level of root resorption (rs = 0.348, *n* = 138, *p* < 0.001). There was no association between infraocclusion and the level of root resorption (*p* = 0.747). Signs of infraocclusion were noted in 32 out of 138 teeth. There was no association between gender and the presence of infraocclusion (*p* = 0.067) and the presence or lack of infraocclusion and the therapeutic status of a tooth (*p* = 0.450). Only 28 out of 138 (20.3%) were intact. There was a significant difference in the level of root resorption between restored and/or carious (median = 3, IQR 2–4) and intact teeth (median = 2, IQR 1.75–3), U = 1168, *p* = 0.044, r = 0.242. **Conclusions**: Overall, 35.2% of corresponding primary molars were present in oral cavity, and were in good condition, and could potentially be preserved in the long term.

## 1. Introduction

Tooth agenesis is one of the most common tooth anomalies, and the second premolar is the most common tooth to be missing [1,2]. This pathology may require orthodontic treatment and sometimes high-cost multidisciplinary future interventions [3].

The choice of treatment depends on several factors such as the number of missing teeth and their position, facial profile, space requirements, skeletal relationship, patient’s attitude towards the treatment, age, and condition of retained primary teeth [4]. Treatment options are orthodontic space closure or space opening for implant placement, prosthodontic treatment, autotransplantation, or no treatment at all if there are no esthetic or functional problems [5]. Many studies focus on the retention of second mandibular primary molars as an agenesis treatment option, evaluating their reliability in the long term [3,4,6,7,8,9,10,11,12,13,14].

The retention of primary molars is a cost-effective and less invasive option than autotransplantation, implant placement, or orthodontic space closure [3]. However, clinicians must take into consideration that retaining primary molars in the oral cavity may lead to their infraocclusion and/or root resorption and subsequent extraction [6]. What needs to be taken into account is that infraoccluded primary molars can be susceptible to several complications, such as the tipping of adjacent teeth, space loss, reduced arch length, midline deviation, and the extrusion of opposite teeth [15].

Some studies suggest the retention of second mandibular primary molars as a viable long-term option [3,6,8,9,10]. The overall prevalence of tooth agenesis in the Latvian population is slightly higher than average in Europe (9.3% and 7%, respectively), making hypodontia management and treatment increasingly important topics in orthodontics [2,16]. Currently, no studies in Latvia have determined the condition of retained second mandibular primary molars in the case of second mandibular premolar hypodontia.

## 2. Materials and Methods

### 2.1. Study Sample

The study sample for the current study was derived from an author’s previous study [16]. In that cross-sectional retrospective study, 2692 panoramic radiographs of adolescent patients were included, focusing on determining the prevalence of hypodontia and any associated dental anomalies. Patients with at least one mandibular 2nd premolar agenesis were included in the current study, providing 156 patients as a study sample. The study sample consisted of 93 females and 63 males, all aged 11 to 14 years old (M = 156 months, SD = 14 months) after excluding those with genetic syndromes in their medical history. Overall, 236 missing permanent mandibular 2nd premolars in 156 patients were recorded. A total of 237 panoramic radiographs were examined.

### 2.2. Panoramic Examination

Panoramic radiographs were taken between August 2020 and September 2021 at the Riga Stradins University Institute of Stomatology (unrelated to this study)—mostly for orthodontic treatment. All radiographs taken before and after the set period for this study were also examined, giving some insight into the survival of retained primary 2nd molars. Out of 156 patients, only 61 had more than 1 panoramic radiograph taken. For these patients, the shortest follow-up was 2.5 months, and the longest was 72 months (M = 27.9 months, SD = 15.2 months).

### 2.3. Radiological Analysis

Panoramic radiographs were retrospectively analyzed in program i-Dixel (Morita, Japan) to determine the presence and condition of retained mandibular primary 2nd molar in the case of the agenesis of a permanent successor. Retained teeth were classified as good or poor based on root resorption, infraocclusion, caries, and restorations. Tooth condition was considered poor if at least 1 of the following was present: (1) ¾ or more of the root was resorbed; (2) an infraocclusion of 0.5 or more; (3) restorations or caries on 3 or more surfaces; and (4) infection-related root resorption.

**Figure 1:**
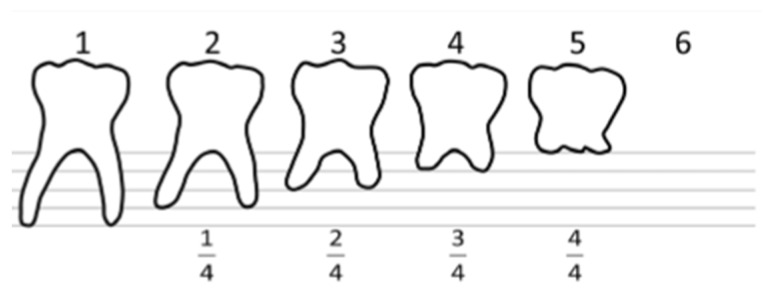
Root resorption evaluation, adapted from Bjerklin and Bennet [6].

Root resorption that was not connected to an infectious process was measured using a method published by Bjerklin and Bennett, 2000 (Figure 1). Each root was scored “1–6”, where “1” means no root resorption and “6” means no tooth left in the oral cavity at all. Mesial and distal roots were measured separately to determine if a more severe resorption level should be given to a tooth.

**Figure 2:**
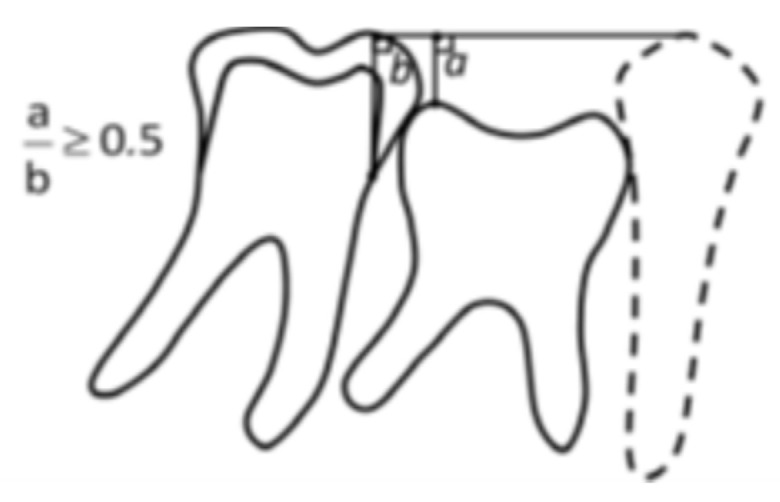
Measurement for infraocclusion, adapted from Hvaring and Birkeland [8].

Infraocclusion was calculated as a ratio, adapted from Hvaring and Birkeland 2020 [8] (Figure 2). It was measured as a ratio between 1st permanent mandibular molar crown height and distance from the second primary mandibular molar occlusal surface and tip of the distal cusp of the permanent molar. An infra-occlusion of 0.2 and more was set as clinically significant since it could be an early sign of ankylosis [17].

**Figure 3:**
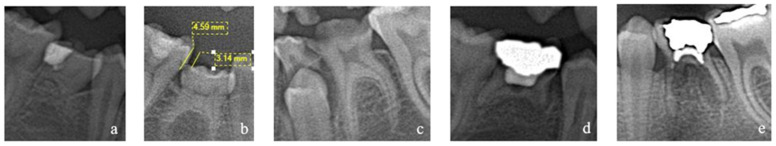
Examples of teeth classified as poor: (**a**) 4/4 of root resorbed; (**b**) infraocclusion of more than 50%; (**c**) caries on 3 or more surfaces; (**d**) restoration on 3 or more surfaces; (**e**) infection-related root resorption.

A separate category for poor prognosis for retained primary teeth was made in this study. Roots were classified as pathologically resorbed if there were signs of rarefying osteitis along with root resorption. Examples of teeth classified as poor are pictured in Figure 3.

### 2.4. Statistical Analysis

For data analysis, the statistical software program Jamovi 2.6.13 (The jamovi project, Australia) was used. Descriptive statistics were used to analyze the data. To find if a statistical association exists between gender and the presence of infraocclusion and infraocclusion and the presence of caries/restorations, the Chi-squared test was used. To analyze any correlation between the level of root resorption and age, Spearman’s correlation test was used. The Mann–Whitney U test was used to find any association between the presence of infraocclusion and the level of root resorption and root resorption and the presence of caries/restorations.

### 2.5. Ethical Committee

This study was approved by Riga Stradins University’s Research Ethics Committee (Nr. 22-2/424/2021) on 10 August 2021.

## 3. Results

The prevalence of mandibular second premolar agenesis was 5.8% (156 patients), with 236 missing teeth. Bilateral agenesis was almost as common as unilateral agenesis (51.3% and 48.7%, respectively). There were no statistically significant differences between genders (*p* = 0.669).

For the set period of this study, 138 (58.5%) predecessor primary molars were present. Further analyzing these 138 teeth shows that 60.1% (N = 83) of them were in good condition. Excluding missing primary molars and those in poor condition leaves only 83 (35.2%) teeth that could be relied on in the long term. A more detailed distribution among conditions and classification is outlined in Table 1. A further analysis of the teeth that are in poor condition shows that the most common reason for being classified in this category is having three-quarters or more of the root resorbed. Table 1 shows that 6.3% of teeth are in poor condition for more than one reason.

The distribution of root resorption and infraocclusion levels in second mandibular primary molars are detailed in Table 2.

Signs of root resorption were visible in patients as young as 132 months (11 years), and at this age, one primary tooth had three-quarters of its root resorbed. There was a statistically significant positive weak correlation between age and the level of root resorption (rs = 0.348, n = 138, *p* < 0.001). The presence of an association between infraocclusion and the level of root resorption was not found (*p* = 0.747).

Some signs of infraocclusion were noted in 32 out of 138 teeth. An infraocclusion of 0.2 or more was reported in 87.5% of molars with infraocclusion and 0.5 or higher in 28.1%. The mean ratio of infraocclusion was 0.36 (min = 0.1; max = 0.73; SD = 0.16). There was no statistically significant association between gender and the presence of infraocclusion (*p* = 0.067). Only 8 out of 32 infraoccluded teeth were completely intact. In general, there was no association between the presence or lack of infraocclusion and the therapeutic status of a tooth (*p* = 0.450). All infraoccluded teeth had both mesial and distal neighboring teeth. Evidence of infraocclusion was detected as early as 11 years old (132 months).

Regarding dental restorations and/or carious lesions, only 28 out of 138 (20.3%) were intact. There was a statistically significant difference in the level of root resorption between restored and/or carious (median = 3, IQR 2–4) and intact teeth (median = 2, IQR 1.75–3), U = 1168, *p* = 0.044, r = 0.242. Out of all retained molars, 17 had signs of pathological root resorption. None of those teeth were intact—eight had pulpotomy, five had caries, three had filling with secondary caries, and one had just a filling.

Out of 156 patients, more than one panoramic X-ray was taken for 61 patients. This gives us 92 hypodontic second premolar teeth whose predecessor primary molars’ condition can be observed over time. The shortest observation period was 2.5 months, and the longest was 72 months (M = 11 months). At baseline, 27.2% (N = 25) of second primary mandibular molars were missing, 33.7% (N = 31) were in poor condition, and 39.1% (N = 36) were in good condition. The data show that 72.2% of teeth whose condition was good (N = 36) stayed in good condition (N = 26). Of all teeth whose condition at the first panoramic X-ray was poor (N = 31) but were present in the oral cavity, 67.7% were missing at the latest panoramic X-ray (N = 21).

## 4. Discussion

The prevalence of second mandibular premolar agenesis in the current sample of the Latvian adolescent population is 5.8%. A total of 236 missing second mandibular premolars were identified. The data from the latest meta-analysis show that the overall prevalence of mandibular second premolars is 3.26% [18], making their prevalence in Latvia higher than the reported overall.

In total, 138 (58.5%) retained second mandibular primary molars were present. Although numerous studies evaluate the persistence and condition of retained primary teeth, only a few report on the primary tooth’s actual presence or absence at baseline. Garib et al., 2014 [19], report that 88% of corresponding primary teeth were present while evaluating second premolar agenesis. In contrast, in the study conducted by Bilinska et al., 2023 [12], only 33% of primary molars were present.

Since the design of this study was a cross-sectional observational study, the emphasis was put on evaluating retained primary molars at one point in time. The presence of additional panoramic X-rays for some patients gave the possibility of assessing the longevity of retained primary molars, although not for a long period. The overall data from this research show that if a primary tooth is in good condition, it mostly stays in good condition (72.2% of the observed teeth), but these data have to be taken with caution because of the short observational period. Regarding the topic of permanent tooth agenesis, great interest is shown in the reliability of retained primary teeth as a treatment option, especially primary molars. From their research in 2000, Bjerklin and Bennett concluded that if a primary tooth is present in the oral cavity at 20 years of age, it has a good prognosis for long-term survival. Several other studies report on the good long-term condition of retained primary molars [7,8,9,10]. Ith-Hansen and Kjæer, 2000 [9], also conclude that the retention of the primary second molar is a viable but semi-permanent solution, adding that there should not be morphological deviations of permanent dentition.

Kjæer et al., 2008 [11] in their research, divided patients into two groups where group I had patients with ectodermal phenotypes (screwdriver-shaped incisors with or without invaginations, slim incisors, taurodontic root morphology, and an unusual agenesis pattern), and group II had patients without any signs seen in group I. They conclude that group I’s retained primary molars do not have the expected long-term persistence [11]. An uncertain long-term prognosis of retained primary second mandibular molars is reported by Bilinska et al., 2023 [12]. In patients older than 17 years, the prognosis of a healthy primary molar is expected to be good but under the condition that it has no distinct infraocclusion or extensive root resorption [12]. A study conducted in the UK also reports conclusions that contrast most Scandinavian studies. It is stated that upper and lower second primary molars have unpredictable life spans—they can be good or poor and are very dependent on an individual [13].

Overall, it has been reported that leaving the primary molar in the oral cavity as a treatment modality is a feasible option since 82–89% of these teeth remain in good condition over a follow-up between 5 and 13 years. Also, root resorption and infraocclusion do not seem to increase substantially [3]. It must be highlighted that in the current research sample, 41.5% of primary teeth were not even present, and 23.3% were in poor condition. This leaves 35.2% of primary teeth in good condition and potentially to be relied upon if the orthodontic management of agenesis is retaining a primary tooth. An important aspect that is not discussed in most of the research papers is caries prevalence in the country of research as it may influence the prognosis and reliability of primary teeth as a treatment option. Bilinska et al., 2023 [12], address this potential bias in their study. The majority of studies that report a good long-term prognosis of retained primary teeth are conducted in Scandinavia, which has one of the lowest caries prevalences in Europe. The D5MFT index of 12 year olds in Latvia is 3.4. In contrast, in Denmark, it is 0.4, and in Sweden, it is 0.8 [20].

The literature shows that physiological root resorption is initiated and regulated by the stellate reticulum and the dental follicle of the successor permanent tooth. The question then arises of how root resorption is also noted in primary teeth without a permanent successor. There is a limited understanding and knowledge of this process. During the growth of an individual, masticatory muscles enlarge, and forces applied to the primary teeth periodontal ligament are too extensive to withstand. This leads to the necrosis of the periodontal ligament, which triggers a cascade of molecular processes leading to root resorption [21]. Severe root resorption (three-quarters and more) was the most common condition among teeth with poor condition in the current study. In a systematic review of the literature, it is stated that root resorption is unpredictable and teeth with more severe resorption are at risk of being lost [14].

A brief literature review highlights the lack of a unified method for determining and measuring the presence and extent of infraocclusion. In the literature, several infraocclusion observation methods exist. Two other studies uses the same method as the current study [4,8]. In other studies, infraocclusion was determined or measured (1) as the distance in tenths of a millimeter between the occlusal line and occlusal surface of a second primary molar [6,7]; (2) as the interval between the occlusal plane and occlusal surface of the second primary molar in millimeters [22]; and (3) absent if the occlusal surface of the primary molar is at the level of the occlusal plane or present if the occlusal surface of the primary molar is below the occlusal plane [19]. Some studies do not specify how the presence of infraocclusion is determined [9,10,23]. The existence of these numerous methods highlights the problem that arises when the overall characteristics of infraocclusion are being investigated between different countries and continents. There is a clear lack of standards that would help determine what level of infraocclusion is clinically significant and needs to be taken into account when planning orthodontic treatment. It has to be taken into account that the same measurement methods used in intraoral X-rays cannot be made in panoramic X-rays because of the slight enlargement of the panoramic image [24].

Bjerklin et al., 2008 [7], and Bjerklin and Bennet, 2000 [6], report similar findings to this study on teeth with signs of infraocclusion—21% and 20.3%. Out of 138 retained primary molars, 9 (6.5%) were infraoccluded by a ratio of 0.5 or more, categorizing them as being in poor condition. A study conducted by Hvaring and Birkeland, 2020 [8], shows that primary molars categorized as in poor condition due to an infraocclusion of more than 0.5 make up 8.8% of all retained primary teeth, which is slightly higher than in this study. In a different study by Hvaring et al., 2014 [4], an infraocclusion ratio of 0.2 or more was set as clinically significant and noted in 43.6% of patients; a ratio of 0.6 was set as extensive and was noted in 18.8% of patients, which was similar to the current study. Other studies that measured infraocclusion just as being present or not present report the presence of it in 64%, 82.3%, and 0% of teeth [10,22,23].

In this study, an association between infraocclusion and the level of root resorption was not found. This is in contrast with two studies that report a strong or significant correlation between root resorption and infraocclusion [4,6].

It is reported that primary mandibular molars are affected by infraocclusion up to 10 times more than primary maxillary molars. Most infraoccluded primary molars with present premolars tend to exfoliate normally, but infraoccluded primary molars without successors usually have slow exfoliation rates [25]. Although present at a low level, there is evidence that infraocclusion does not affect the lifespan of retained primary molars in the case of premolar agenesis, especially in older patients [3].

All 17 teeth that had inflammatory root resorption had pulpotomy, fillings, and/or caries. Statistically significant associations with inflammatory root resorption and gender, age, and the therapeutic status of the tooth have been reported in another study [26]. The current study does not provide enough data to carry out statistical tests.

## 5. Conclusions

This study finds that the majority of second mandibular primary molars were either already missing or in poor condition. For these children, some level of intervention may be required, which could be costly, possibly involving several fields of dentistry. Caries prevalence may be an impacting factor on primary molar prognosis and must be taken into consideration while planning the orthodontic treatment. There was a weak association between age and the level of root resorption. A stronger association between root resorption and the therapeutic status of the tooth was found.

## Figures and Tables

**Table 1:** Condition of retained primary 2nd mandibular molars.

CONDITION	N	%
**Good condition**	**83**	**35.2%**
**Not present in the oral cavity**	**98**	**41.5%**
**Poor condition** - ¾ or more of the root resorbed - An infraocclusion of 0.5 or more - Restorations or caries on 3 or more surfaces - Infection-related root resorption - More than one of the reasons mentioned above	**55** 16 8 5 11 15	**23.3%** 6.8% 3.4% 2.1% 4.7% 6.3%
**Total**	**236**	**100%**

**Table 2:** Distribution of root resorption and infraocclusion levels in 2nd mandibular primary molars. **NI**—no infraocclusion or so minimal that it cannot be measured using a panoramic X-ray; **CBE**—cannot be evaluated due to being too restored or carious to find measurement points.

**Root Resorption**							
	**1**	**2**	**3**	**4**	**5**	**6**	**Pathological**
**Number of teeth (Total = 236)**	29	35	33	16	8	98	17
**Infraocclusion**							
	**NI**	**<0.20**	**0.21–0.50**		**>0.50**		**CBE**
**Number of teeth (Total = 136)**	100	5	19		8		4

## Data Availability

Data are available on request.
